# Mommio’s Recipe Box: Assessment of the Cooking Habits of Mothers of Preschoolers and Their Perceptions of Recipes for a Video Game

**DOI:** 10.2196/games.8142

**Published:** 2017-10-17

**Authors:** Maciel Ugalde, Leah Brand, Alicia Beltran, Hafza Dadabhoy, Tzu-An Chen, Teresia M O'Connor, Sheryl O Hughes, Tom Baranowski, Richard Buday, Theresa A Nicklas, Janice Baranowski

**Affiliations:** ^1^ Childrens Nutrition Research Center Department of Pediatrics Baylor College of Medicine Houston, TX United States; ^2^ School of Information Center for Health Communications Research University of Michigan Ann Arbor, MI United States; ^3^ Health Research Institute University of Houston Houston, TX United States; ^4^ Archimage, Inc Houston, TX United States

**Keywords:** parenting, video game, child, preschool, cooking, vegetables

## Abstract

**Background:**

Vegetables are an important part of a healthy diet because they help prevent several chronic diseases. Mothers of preschoolers reported difficulty getting their young children to eat vegetables, and many did not know how to cook child-pleasing recipes.

**Objective:**

The cooking habits of mothers of preschoolers, their perceptions of recipes designed for their children, and the involvement of their children in food preparation were assessed to inform a food parenting video game called *Mommio*.

**Methods:**

A cross-sectional survey design was used. Eligibility criteria included mothers of 3- to 5-year-old children who reported difficulty getting their children to eat vegetables. Participants completed a demographic questionnaire with questions about their food preparation practices. They were asked to select up to 4 of the 10 provided recipes they wanted to try and to prepare and report back on their experiences.

**Results:**

Most (46) of the 50 recipes included in *Mommio’s* in-game recipe box were evaluated at least once and some up to 5 times with a total of 85 evaluations. This well-educated, mostly employed, sample of 27 mothers of preschoolers preferred simple, quick recipes. They ate primarily at home, made dinners from scratch, and indicated that the 46 recipes were generally simple, quick, and easy to prepare. Involvement in preparation enhanced their child’s acceptance of the food. Prior food and preparation preferences influenced the children’s acceptance of the dish at the ensuing meal.

**Conclusions:**

The high rate of home recipe preparation indicated that including a recipe selection and preparation component in a food parenting video game could be attractive and may enhance effectiveness. Mothers reported that the recipes provided were generally easy to prepare, tasted good, and the instructions were easy to understand, suggesting they could be helpful to the mothers when playing a vegetable parenting game. Some mothers reported that involving their children in recipe preparation influenced their children’s willingness to eat the vegetables. The highest rated recipes are being included in the game, and mothers will be encouraged to involve their children in recipe preparation.

## Introduction

Vegetables are an important part of a healthy diet because they help prevent several chronic diseases [1,2]. As childhood eating habits are initiated early and tend to track into adulthood [3], young children should eat recommended amounts of vegetables to establish a lifelong pattern for optimal health. However, many mothers reported difficulty getting their young children to eat vegetables, and many did not know how to cook child-pleasing recipes [4]. Although research has found that levels of food preparation skills were related to a higher quality of dietary intake, the literature is inconsistent about how frequently adults prepare food at home. In one study, most young adults (males and females) performed food preparation tasks monthly or less often [5], whereas in another study, two-thirds reported cooking the main meal at least 5 times per week [6]. Other studies found that elementary school students with higher self-efficacy for cooking were more likely to consume a family dinner [7] compared with those with higher involvement in food preparation [8]. In Wales, some parents learned cooking skills at a university or in community cooking classes [9], but new technologies are now available that may distribute cooking education more widely [10]. How frequently any particular group of parents, who might use new technologies, is likely to cook and thereby be able to benefit from training in recipe preparation is not clearly known.

*Mommio*, a first-person role-playing mobile three-dimensional (3D) video game currently under development [11,12], is being designed to train players in parenting practices effective in getting children to enjoy and eat vegetables over the long term [13]. Intended for mothers of preschool-aged children, an alpha test of an early version of *Mommio* featured an in-game “recipe box” containing a limited number of vegetable recipes [11]. Participating mothers reported that they liked the feature, requested that more recipes be provided in the game, and said that they wanted to try the recipes in real life [11]. Although a recipe feature would appear desirable in *Mommio*, some studies have indicated a high frequency of eating meals away from home, thereby possibly negating the value of a recipe feature [14].

Interactive media, including video games, have been shown to advance parenting skills [15]. Although a recent review of cooking interventions had positive outcomes, the preferred design of cooking or food preparation interventions for inclusion in a video game could not be determined because of their substantial heterogeneity [16]. Although studies have indicated that involving children in preparation enhanced children’s acceptance of or preference for the foods [17,18], this has not been demonstrated with preschoolers. Involvement of children in setting goals to make recipes increased fruit and vegetable consumption [19] but was limited to older children. Previous studies have assessed cooking skills of individuals and tested recipes for a specific audience. One study assessed the self-reported confidence using eight cooking techniques, confidence in cooking ten foods, and ability to prepare four types of dishes in individuals aged 19 years or older [20]. Another study described the methods followed to create a Web-based cookbook, “@TheTable,” to provide pediatric cancer patients and survivors resources to lead healthier lifestyles [6]. However, these studies did not necessarily generalize to mothers of healthy young children. Furthermore, the types of recipes that appeal to mothers of preschoolers are not clearly known; so, this study asked mothers about their preferences.

This study assessed the following: (1) the cooking habits of mothers of preschoolers who had difficulties getting their children to eat vegetables, (2) the acceptability and usefulness of potential recipes in a vegetable parenting game (ie, *Mommio*), and (3) whether mothers would report that involving their preschool children in food preparation would increase their children’s consumption of vegetables. Participants also evaluated recipes designed for possible inclusion in the game.

## Methods

### Research Design

A cross-sectional Web-based questionnaire of parents of 3- to 5-year-old children was followed by testing and evaluating recipes; the recipe evaluations were reported in an online questionnaire. Eligible participants included mothers who reported difficulty getting their children to eat vegetables. Mothers who reported no difficulty with vegetables or noted that their children did not live with them most of the time were excluded. Participants were recruited from a panel of mothers who had participated in prior formative research for the *Mommio* game, supplemented with additional participants recruited from the Children’s Nutrition Research Center’s research volunteer list, social media posts, and flyers posted throughout Houston’s Texas Medical Center and Rockford, Michigan. The sample was kept to a small number to facilitate the speed of data collection and analysis for game development. Saturation, the point at which no additional responses are reported, is the usual criterion for sample size in qualitative research [21]. In our experience of qualitative research with parents on children’s diet, a sample of 20 is ample to reach saturation. This study, including its methods and questionnaire items, was approved by the institutional review board of the Baylor College of Medicine. Digital informed consent was obtained.

### Recipes

A list of 10 vegetables (potato, broccoli, pepper, corn, carrot, tomato, zucchini, spinach, green beans, and cucumber) most frequently consumed by 3- to 5-year-olds was generated from the National Health and Nutrition Examination Survey. Recipes that included these and many other nutritious vegetables were collected primarily from our prior projects and public websites [22-25]. Given the palates of small children and the assumption that mothers may have limited time and cooking experience, recipes were selected with simplicity in taste and preparation in mind. Modifications were made, such as removing salt and pepper amounts from the ingredient list and substituting “as per taste.” Ingredients not commonly found at grocery stores or deemed expensive were removed or modified with more commonly available pantry items or less expensive alternatives. The main nutritional consideration when selecting recipes was to provide 4 vegetable servings of half a cup each. No explicit restrictions were placed on the nutrient composition of the recipes but those high in fat and sugar were not included. Recipes included side dishes made to accompany a larger caloric item and main dishes designed to be the meal’s major energy source. Listings of ingredients and preparation instructions for the 50 recipes are available from the corresponding author. To facilitate selection for testing, recipes were combined into 5 groups such that each group of 10 recipes contained side dishes, main dishes, and a variety of vegetables.

### Game Design

**Figure 1:**
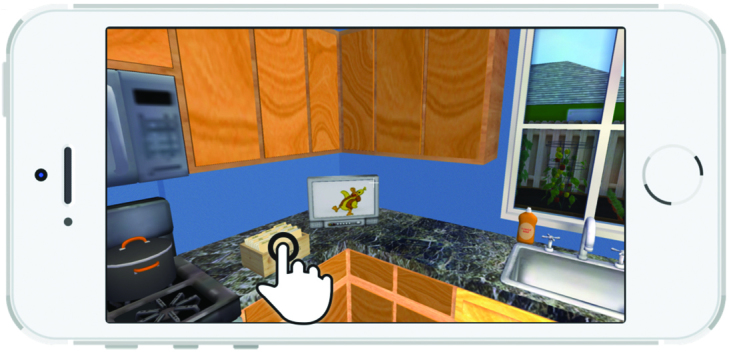
Initiate food preparation.

**Figure 2:**
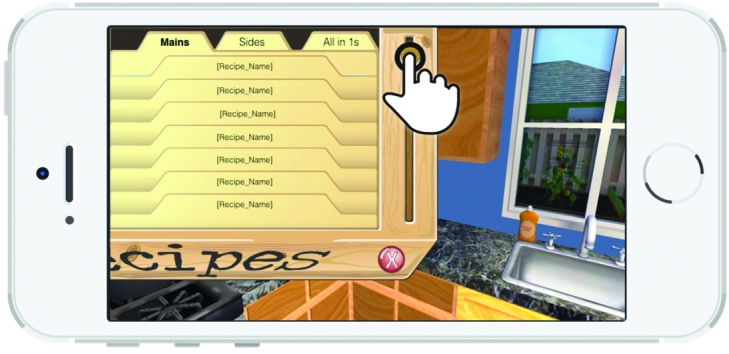
Recipe box.

**Figure 3:**
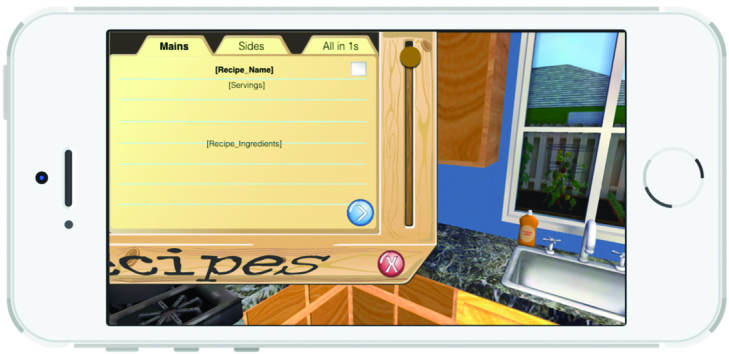
Recipes.

**Figure 4:**
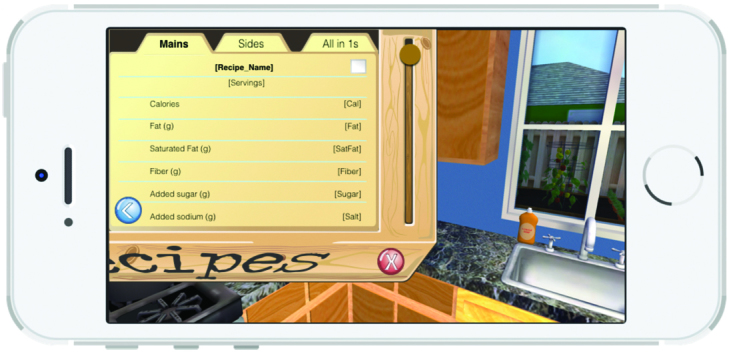
Nutrient content of recipes.

A 3D representation of a home and a grocery store allows a child character (nicknamed “Kiddio”), a dog, and a male adult character to roam the house. The nonplayer characters are controlled by artificial intelligence algorithms. The player assumes the role and point of view of the child’s mother. Player exploration (walking around, opening cabinets, operating kitchen appliances, etc) is permitted and expected. The player primarily interacts with Kiddio, a preschool-aged character, who prompts the player to act by saying “I’m hungry.” As shown in Figure 1, the player must then select and prepare a vegetable recipe in the kitchen’s recipe box on which the mother must click. The recipes were organized into three categories. When the player selects a category, a series of recipe names appear (Figure 2). When a specific recipe is clicked, the recipe name, numbers of servings, and ingredients appear (Figure 3). Another click elicits the nutrient content of the recipe (Figure 4). Once all four cards are seen, the player is informed that the dish is now prepared and placed on the kitchen table. The player mother needs to have vegetables at home to prepare the recipes. If no vegetable is available at home, the mother must go to the store to purchase some. She gets points for bringing the child to the store and for involving the child in recipe preparation. Once the player and Kiddio are seated at the kitchen table, the player encourages Kiddio to eat the vegetable, but Kiddio refuses. The player can then try different food parenting strategies by choosing from four statements to tell Kiddio—two effective or two ineffective—or modifying the environment (eg, turning off the television). If she selects ineffective ways, she moves toward losing the game. Selecting effective parenting items moves the player toward winning. At the conclusion of each *Mommio* quest, Kiddio either tastes the vegetable to signify a victory or runs out of the room, indicating a loss.

### Data Collection

Each participant first completed a demographic and screening questionnaire that included the questions listed in Tables 1 and 2. Those who qualified were randomly assigned to one of 5 recipe groups. Every participant was emailed an invitation with a link to an online recipe selection questionnaire, unique to her assigned group. Then, participants selected up to 4 of the 10 recipes to prepare, serve to, and evaluate with their child. After recipe selection, participants received an individualized email with instructions and copies of their selected recipes. Upon recipe completion, participants were directed to an online evaluation questionnaire, which included open-ended questions about the prepared recipes and whether and how the child was involved in cooking. A sample of 20 parents participated in the evaluation of the recipes. Another 7 did not respond to email inquiries after completing the screener. A total of 7 parents volunteered to evaluate more than their original 4 recipes. Participants received gift cards for completing each component. Participants who tested and evaluated 4 or more recipes also received a kitchen apron containing a *Mommio* video game logo. To ensure most recipes were tested, the last 4 participants to join the study were asked to include recipes not selected by previous participants. Testers were obtained for 46 of the 50 recipes. An overall recipe rating scale was employed: “Considering your entire experience of preparing, serving, and tasting the recipe, how would you rate this recipe?” with the following 5 response options: “Hated it,” “Disliked it,” Didn’t hate or like it,” “Liked it,” or “Loved it.”

### Data Analysis

Quantitative data were analyzed using the statistical analysis system (SAS version 9.4, SAS Inc, Cary, NC). Descriptive statistics were used to calculate frequencies and percentages. Answers to open-ended questions were coded using thematic analysis [26] according to the following themes: affordability of ingredients, recipe instructions and preparation, healthy meals, children’s involvement in cooking, sensory evaluation, and recipe modifications. Thematic analysis lists categories of similar comments obtained from the transcript within prespecified themes, which defined the purpose of the study. In contrast to more formative research, which clusters comments and redefines clusters across multiple iterations, thematic analysis is primarily a list of nonduplicated responses within prespecified categories.

## Results

### Sample Descriptors

A nonrepresentative sample of 27 mothers of 3- to 5-year-old children participated in the study. They included mothers who had contributed to prior formative research for the *Mommio* game (11 participants), were selected from the Children’s Nutrition Research Center’s research volunteer list (11 participants), or were recruited via online posts and flyers (5 participants). The sample was well educated (70% had graduated from college), married (78%), employed (78%), worked full-time outside the home (63%), and had a household income over US $60,000/year (59%; Table 1).

**Table 1:** Sample demographic characteristics for selection survey (N=27).

Characteristics		n (%)
**Child’s gender**		
	Male	20 (74)
	Female	7 (26)
**Education**		
	High school graduate or general equivalency diploma	1 (4)
	Technical school	1 (4)
	Some college	6 (22)
	College student	7 (26)
	Postgraduate study	12 (44)
**Marital status**		
	Married or living with someone else	21 (78)
	Single, never married	4 (15)
	Divorced, separated, or widowed	2 (7)
**Employed**		
	Yes	21 (78)
	No	6 (22)
**How do you usually spend your day?**		
	I work full time outside the home	17 (63)
	I work full time at home for pay	0 (0)
	I work part time outside the home	3 (11)
	I work part time at home for a pay	0 (0)
	I am a student	0 (0)
	I am a stay-at-home mom	7 (26)
**Income**		
	Less than US $30,000	5 (19)
	US $30,000 to US $60,000	6 (22)
	Over US $60,000	16 (59)

Most of these mothers (52%) made lunches for their children on weekends only and reported diverse frequencies of preparing snacks for their children but provided dinner or supper at home most days of the week (59%; Table 2). Most mothers (63%) reported the ability to cook, but preferred simple, quick recipes; finding quick, easy ways to make foods for lunches (78%); preparing finger foods (44 %) or fruit and vegetables (41%) for snacks; and cooking from scratch for dinner (63%; Table 2).

**Table 2:** Food preparation skills and practices from the screening survey (N=27).

Survey questions		n (%)
**How would you rate your cooking ability?**		
	I am an excellent cook and love to cook from scratch	4 (15)
	I can cook but prefer to use very simple, quick recipes	17 (63)
	I can cook when I have to, but I don’t like to cook	4 (15)
	I can cook if I use mostly premade dishes or mixes	0 (0)
	I can cook a little, but I’m not very confident in my kitchen skills	2 (7)
	I can’t cook	0 (0)
**How often do you provide lunch at home for your 3- to 5-year-old child?**		
	Post 7 days/week	5 (19)
	Most days of the week	6 (22)
	Weekends only	14 (52)
	None	2 (7)
	Other	0 (0)
**When you provide lunch at home for your 3- to 5-year-old child, what best describes what you do?**		
	Make a meal mostly from scratch using various recipes	5 (19)
	Find quick, easy to make foods (eg, salads, leftovers, sandwiches, and simple recipes)	21 (78)
	We eat a lot of fast food or deli take-out, something quick	1 (4)
**How often do you provide a snack at home for your 3- to 5-year-old child?**		
	Several times a day, every day	8 (30)
	Once a day, 7 days/week	8 (30)
	Most days of the week	8 (30)
	Weekends only	3 (11)
	None	0 (0)
	Other	0 (0)
**When you provide a snack at home for your 3- to 5-year-old child, what best describes what you do?**		
	I serve finger foods, mostly food other than fruit and vegetables (eg, prepackaged snacks, lunchables, and heat and eat type of foods)	12 (44)
	I serve mostly fruit and vegetables	11 (41)
	I use recipes to make snacks from scratch	0 (0.0)
	Other	4 (15)
**How often do you provide dinner or supper at home for your 3- to 5-year-old child?**		
	7 days/week	10 (37)
	Most days of the week	16 (59)
	Weekends only	0 (0)
	None	0 (0)
	Other	1 (4)
**When you provide dinner, supper, or the evening meal at home for your 3- to 5-year-old child, what best describes what you do?**		
	I make a meal mostly from scratch using various recipes	17 (63)
	I find quick, easy to make foods (eg, meal mixes, preprepared foods from restaurants or grocery store, and heat and serve foods)	6 (22)
	We eat a lot of fast food or restaurant take-out, something quick and easy	2 (7)
	Other	2 (7)

### Recipe Ratings

A subsample of 20 mothers evaluated a total of 46 recipes. Each recipe was evaluated at least once and up to 5 times with a total of 85 evaluations of the 46 recipes. The recipes were well received. A total of 13 recipes received a mean recipe rating of 5 (“Loved it”), with another 13 receiving a 4 or higher rating (“Liked It”). Of these top 26 recipes, 20 recipes were rated as “very easy to prepare” and another 8 recipes rated as “somewhat easy.” A diverse variety of vegetables were rated at the higher end of the mean recipe rating scale. The same diversity was true for the vegetables at the lower end of the scale, suggesting no one or two vegetables dominated the positive or negative ratings. Furthermore, 4 recipes were not selected for testing by at least one mother. No obvious pattern can be discerned as to why these were not selected, except possibly for “spiciness” in the title of one recipe. Neither the number of ingredients nor the reported ease of preparation appeared to be associated with the recipe ratings. As would be expected, the taste of the recipe was related to the mean overall recipe rating, and the instructions were generally easily understood.

Less than half of the parents (45%) had prepared a similar dish before. When asked how likely it was that they would make each dish, 46% of the parents reported that the dishes would definitely be made and 35% reported that they might be made. In addition, 61% of the parents indicated they had all of the ingredients at home needed to prepare the recipes. Moreover, 74% of the parents reported that the recipe instructions were “very easy to follow with few ingredients” and easy to prepare (87%):

My family loves chick peas and hummus; it is pricey to buy it already made. I always wanted to learn how to make it, but I thought it was difficult, but this recipe proved me wrong.

Many reported that the ingredients were inexpensive:

Peppers have great benefits and usually they are on sale.

Most parents (81%) liked the finished recipes. Parents mentioned that they enjoyed learning new ways to prepare vegetables:

A different way of preparing veggies is always nice.

Other parents appreciated the new take on old recipes:

It was easy and something similar to what I have made before, just without lemons.

Multiple parents evaluated the recipes according to the time needed for preparation or expressed a concern about long preparation time:

It was simple and did not take a lot of time to cook.

The recipe was very easy to follow. It just takes a lot of time.

The preparation took longer than I expected.

One of the mothers commented on the need for only basic equipment:

I really liked this recipe. It was different and very easy. Love that I only had to use one pot.

Alternatively, another mother commented on the food preparation skills needed to prepare one of the recipes:

It was extremely difficult to cut the squash. It was hard and difficult to control or hold still. The deseeding was like trying to clean out a pumpkin—very messy.

Some parents said they liked that the recipes were a healthier version of some foods they already consumed:

I liked it, it was easy to make. It’s a healthier substitution for any dip...

It is a great recipe. I usually hate the salty pizzas from stores although we have to eat them once in a while. The recipe is simple and I can add salt as little as I want.

### Child Involvement in Recipe Preparation

The 3- to 5-year-old children were involved in preparing 51% of the recipes. One of the participants stated:

It was easy to do with the kids, very few ingredients all in one baking dish. They enjoyed the experience.

Participants indicated that children helped by washing vegetables; adding ingredients to the blender, bowls, or pots; or measuring or mixing ingredients. Some participants commented on how children’s involvement in the preparation of the recipes influenced their willingness to try the foods:

She was very proud to serve what she made and she made her siblings excited about helping with meals as well.

...did not want to try them but since she helped making the recipe and the 3-year-old ate all of his, she went ahead and ate them once I gave her ranch yogurt dressing to dip them in.

### Child Consumption of Recipe

Approximately 88% of the children tasted the finished recipes and 73% ate them. Prior food preferences were mentioned as an important factor when children decided to try their food:

He doesn’t dislike broccoli so it wasn’t hard to ask him to try.

They hate the taste of pepper, so I was not able to make them eat even a bite.

Some mothers mentioned how the method of preparation influenced their children’s consumption of the recipe:

Both of my children enjoyed the recipe more than I expected. The small cubes of potatoes really helped them eat it better.

My child had a hard time handling the corn and eating it off the cob.

The final presentation of the recipe was also relevant:

It was a very different pasta recipe for him, he loved that it had corn and beans and it is very colorful.

He didn’t want to try it at all, maybe because it looked boring

Spicy flavors were not popular among 3- to 5-year-old children:

For my little people, cayenne pepper is not a spice they prefer, so I think it’s an odd seasoning in this recipe. I even cut it in half and still had complaints about being too spicy...

Another mother mentioned:

Kids were afraid to try at first because they thought it would be spicy.

### Recipe Modification

Only 40% of the participants modified the different recipes when evaluating them. These modifications were made because of the family food preferences. They commented:

We don’t like the taste of parsley so I used a purple leaf...don’t know the name of it but it is my 3-year-old’s favorite vegetable growing in our backyard.

We added olives. We always add olives to the pizza. My kids insist.

When asked if they would modify the recipe before they make it again, 54% said they would:

I would add something to make it more flavorful. The red peppers were good and the lemon juice, but even after adding salt and pepper I would have added more spices or other veggies to make it have a more flavorful taste.

The small potatoes I bought for the recipe inspired me. I want to try to make them tastier.

## Discussion

### Principal Findings

This sample of 27 well-educated and mostly employed mothers of preschoolers regularly cooked at home, unlike most families in the United States [14]. Although they tended to make and serve quick, easy dishes for lunches and snacks, they made dinner from scratch and infrequently ate out of their home. The high frequency of home-prepared meals in this sample (22% and 59% of mothers who participated in this study said that they prepared lunch and dinner at home, respectively, most days of the week) was similar to that in a population-based study in the United Kingdom [6]. This may indicate that mothers interested in playing a video game to promote effective vegetable parenting practices prepare a lot of meals offered to their preschool child and, thereby, could benefit from a recipe preparation component in the game.

Of the 50 recipes offered, 46 were tested by at least one mother. A total of 41 recipes received scores above the median possible recipe score (2.5), with 13 receiving the highest rating (5) and 18 receiving the next highest rating (4-4.5). This suggests that an ample number of tasty, easy-to-understand and prepare recipes are available for incorporating into *Mommio*. According to open-ended questionnaire responses, mothers preferred recipes that included basic ingredients and little equipment, similar to a review about recipe development for limited-resources audiences [27]. Mothers also enjoyed recipes from the app that showed different ways to prepare and present vegetables to their children.

Some participants (15%) reported that the recipes were healthier versions of foods they currently ate. However, mothers’ primary perceived barrier to preparing the recipes was the time required. In 2015 in the United States, 59% of all women and 70% of mothers with children under the age of 18 years were employed outside their home [28], similar to the sample in this study. To overcome the struggle of making time-efficient and nutritious family meals, working women often incorporated precooked and convenience foods into their daily family meals [29] and preferred preparing an evening meal in less than 15 min [30]. Attempting to prepare meals quickly could lead to a decreased transfer of cooking skills from parents to youth, with the inability of children to prepare healthy meals as they transition to adulthood (as identified among young adults) [5].

Older children (aged 6-10 years) who participated in the food preparation process ate significantly more salad 42 g (76%) than the ones where only the parent prepared, likely because of enhanced feelings of autonomy and pride [31]. Mothers in this study perceived that their children enjoyed being involved in the preparation of the recipes and increased their willingness to try the foods similar to other findings [27]. As only half of the parents involved their children, future research should address factors influencing child involvement in home food preparation.

The mothers in this study mentioned that the way ingredients were presented was an important factor in a kid friendly recipe. The need to cut food into 0.5-inch chunks to avoid choking hazards [32] was well received by their children. Future versions of the recipe box app will include warnings to ensure child safety when tasting the recipes.

Research has indicated that preexisting child food preferences were a major determinant of intake [17]. In this study, participants were randomly assigned to one of the 5 different groups containing 10 recipes of a variety of dishes and vegetables to decrease selection bias. Although recipes met other needs of the parent and child, children tended to eat the foods they already preferred. This suggests that parents should know their children’s preferences and make and serve vegetables generally congruent with those preferences, along with occasionally introducing new vegetables. Other strategies available for increasing children’s acceptance of low preference foods include offering the child choices, modeling, involving the child in food purchases and preparation, among others [33,34].

### Limitations

As the game’s name suggests, *Mommio* was designed for mothers, and therefore, fathers were not included in this sample. No effort was made to deliberately include mothers with high school education or less, a demographic known to lack confidence in preparing healthy meals [35]. Future studies should be designed to address this harder-to-reach group of mothers. To increase participant diversity, recruitment should be attempted at community centers, food banks, and day care. Moreover, the study’s design and recruitment procedures may have self-selected mothers who like to cook. All data were obtained by self-report from the mothers, who could be biased, and the sample was small, which limits internal validity and generalizability. However, this was an efficient method for collecting and analyzing data rapidly enough to influence the game design.

### Conclusions

We found that the high rate of home recipe preparation indicated that including a recipe selection and preparation component in a food parenting video game could be attractive and may enhance effectiveness. Mothers reported that the recipes provided were generally easy to prepare, tasted good, and the instructions were easy to understand, suggesting they could be helpful to the mothers when playing a vegetable parenting game. Some mothers reported that involving their children in recipe preparation influenced their children’s willingness to eat the vegetables. The highest rated recipes are being included in the game, and mothers will be encouraged to involve their children in recipe preparation.

## Abbreviations

3D: three-dimensional
USDA/ARS: United States Department of Agriculture/Agricultural Research Service

## References

[1] Andres S, Abraham K, Appel KE, Lampen A (2011). Risks and benefits of dietary isoflavones for cancer. Crit Rev Toxicol.

[2] Boeing H, Bechthold A, Bub A, Ellinger S, Haller D, Kroke A, Leschik-Bonnet E, Müller MJ, Oberritter H, Schulze M, Stehle P, Watzl B (2012). Critical review: vegetables and fruit in the prevention of chronic diseases. Eur J Nutr.

[3] Craigie AM, Lake AA, Kelly SA, Adamson AJ, Mathers JC (2011). Tracking of obesity-related behaviours from childhood to adulthood: a systematic review. Maturitas.

[4] Hayter AK, Draper AK, Ohly HR, Rees GA, Pettinger C, McGlone P, Watt RG (2015). A qualitative study exploring parental accounts of feeding pre-school children in two low-income populations in the UK. Matern Child Nutr.

[5] Larson NI, Perry CL, Story M, Neumark-Sztainer D (2006). Food preparation by young adults is associated with better diet quality. J Am Diet Assoc.

[6] Adams J, Goffe L, Adamson AJ, Halligan J, O'Brien N, Purves R, Stead M, Stocken D, White M (2015). Prevalence and socio-demographic correlates of cooking skills in UK adults: cross-sectional analysis of data from the UK National Diet and Nutrition Survey. Int J Behav Nutr Phys Act.

[7] Woodruff SJ, Kirby AR (2013). The associations among family meal frequency, food preparation frequency, self-efficacy for cooking, and food preparation techniques in children and adolescents. J Nutr Educ Behav.

[8] Leech RM, McNaughton SA, Crawford DA, Campbell KJ, Pearson N, Timperio A (2014). Family food involvement and frequency of family dinner meals among Australian children aged 10-12years. Cross-sectional and longitudinal associations with dietary patterns. Appetite.

[9] Khanom A, Hill RA, Morgan K, Rapport FL, Lyons RA, Brophy S (2015). Parental recommendations for population level interventions to support infant and family dietary choices: a qualitative study from the Growing Up in Wales, Environments for Healthy Living (EHL) study. BMC Public Health.

[10] Adam M, Young-Wolff KC, Konar E, Winkleby M (2015). Massive open online nutrition and cooking course for improved eating behaviors and meal composition. Int J Behav Nutr Phys Act.

[11] Brand L, Beltran A, Buday R, Hughes S, O'Connor T, Baranowski J, Dadabhoy HR, Diep CS, Baranowski T (2015). Training vegetable parenting practices through a mobile game: iterative qualitative alpha test. JMIR Serious Games.

[12] Baranowski T, O'Connor T, Hughes S, Beltran A, Baranowski J, Nicklas T, Sleddens E, Thompson D, Lu AS, Buday R, Arnab S, Dunwell I, Debattista K (2012). Smart phone video game simulation of parent-child interaction: Learning skills for effective vegetable parenting. Serious Games for Healthcare: Applications and Implications.

[13] Baranowski T, Chen TA, O'Connor T, Hughes S, Beltran A, Frankel L, Diep C, Baranowski JC (2013). Dimensions of vegetable parenting practices among preschoolers. Appetite.

[14] Robson SM, Stough CO, Stark LJ (2016). The impact of a pilot cooking intervention for parent-child dyads on the consumption of foods prepared away from home. Appetite.

[15] Annaim A, Lassiter M, Viera AJ, Ferris M (2015). Interactive media for parental education on managing children chronic condition: a systematic review of the literature. BMC Pediatr.

[16] Hersch D, Perdue L, Ambroz T, Boucher JL (2014). The impact of cooking classes on food-related preferences, attitudes, and behaviors of school-aged children: a systematic review of the evidence, 2003-2014. Prev Chronic Dis.

[17] Mazarello Paes V, Ong KK, Lakshman R (2015). Factors influencing obesogenic dietary intake in young children (0-6 years): systematic review of qualitative evidence. Br Med J Open.

[18] Cunningham-Sabo L, Lohse B (2014). Impact of a school-based cooking curriculum for fourth-grade students on attitudes and behaviors is influenced by gender and prior cooking experience. J Nutr Educ Behav.

[19] Cullen KW, Watson KB, Zakeri I, Baranowski T, Baranowski JH (2007). Achieving fruit, juice, and vegetable recipe preparation goals influences consumption by 4th grade students. Int J Behav Nutr Phys Act.

[20] Li R, Raber M, Chandra J (2015). Developing a healthy web-based cookbook for pediatric cancer patients and survivors: rationale and methods. JMIR Res Protoc.

[21] Fusch PI, Ness LR (2015). Nsuworks.nova.edu.

[22] United States Department of Agriculture Fns.usda.

[23] Geniuskitchen.

[24] Allrecipes.

[25] Foodnetwork.

[26] Braun V, Clarke V (2006). Using thematic analysis in psychology. Qual Res Psychol.

[27] Reed DB, Schuster E (2002). Recipe checklist: a tool to aid development of recipes for audiences with limited resources. J Ext.

[28] Hess C, Milli J, Hayes J, Hegewisch A, Mayayeva Y, Román S, Anderson J, Augeri J (2015). Statusofwomendata.

[29] van der Horst K, Brunner TA, Siegrist M (2011). Ready-meal consumption: associations with weight status and cooking skills. Public Health Nutr.

[30] Soliah LA, Walter JM, Jones SA (2012). Benefits and barriers to healthful eating. What are the consequences of decreased food preparation ability?. Am J Lifestyle Med.

[31] van der Horst K K, Ferrage A, Rytz A (2014). Involving children in meal preparation. Effects on food intake. Appetite.

[32] Izumi-Taylor S, Rike C (2011). Prepare healthy foods with toddlers. Dimens Early Child.

[33] Nixon CA, Moore HJ, Douthwaite W, Gibson EL, Vogele C, Kreichauf S, Wildgruber A, Manios Y, Summerbell CD, ToyBox-study group (2012). Identifying effective behavioural models and behaviour change strategies underpinning preschool- and school-based obesity prevention interventions aimed at 4-6-year-olds: a systematic review. Obes Rev.

[34] Peters J, Parletta N, Campbell K, Lynch J (2013). Parental influences on the diets of 2- to 5-year-old children: systematic review of qualitative research. J Early Child Res.

[35] Driver SC, Friesen CA (2016). Impact of a pilot intervention to improve nutrition knowledge and cooking confidence among low-income individuals. J Food Res.

